# Modelling the temporal interplay between stress and affective disturbances in pathways to psychosis: an experience sampling study

**DOI:** 10.1017/S0033291720004894

**Published:** 2022-10

**Authors:** Annelie Klippel, Anita Schick, Inez Myin-Germeys, Christian Rauschenberg, Thomas Vaessen, Ulrich Reininghaus

**Affiliations:** 1 Department of Neurosciences, Center for Contextual Psychiatry (CCP), KU Leuven, Leuven, Belgium; 2 Department of Psychiatry and Neuropsychology, School of Mental Health and Neuroscience, Maastricht University, Maastricht, The Netherlands; 3 Department of Lifespan Psychology & Department of Methods and Statistics, Faculty of Psychology and Educational Sciences, Open University, The Netherlands; 4 Department of Public Mental Health, Central Institute of Mental Health, Medical Faculty Mannheim, Heidelberg University, Heidelberg, Germany; 5 Health Service and Population Research Department, Centre for Epidemiology and Public Health, Institute of Psychiatry, Psychology & Neuroscience, King's College London, London, UK; 6 ESRC Centre for Society and Mental Health, King's College London, London, UK

**Keywords:** Ecological Momentary Assessment, minor daily stress, psychotic experiences, affective disturbance, first-degree, relatives, psychosis

## Abstract

**Background:**

One putative psychological mechanism through which momentary stress impacts on psychosis in individuals with increased liability to the disorder is via affective disturbance. However, to date, this has not been systematically tested. We aimed to investigate whether (i) cross-sectional and temporal effects of momentary stress on psychotic experiences via affective disturbance, and (ii) the reverse pathway of psychotic experiences on stress via affective disturbance were modified by familial liability to psychosis.

**Methods:**

The Experience Sampling Method was used in a pooled data set of six studies with three groups of 245 individuals with psychotic disorder, 165 unaffected first-degree relatives, and 244 healthy control individuals to index familial liability. Multilevel moderated mediation models were fitted to investigate indirect effects across groups cross-sectionally and multilevel cross-lagged panel models to investigate temporal effects in the proposed pathways across two measurement occasions.

**Results:**

Evidence on indirect effects from cross-sectional models indicated that, in all three groups, effects of stress on psychotic experiences were mediated by negative affect and, *vice versa*, effects of psychotic experiences on stress were mediated by negative affect, with all indirect effects being weakest in relatives. Longitudinal modelling of data provided no evidence of temporal priority of stress in exerting its indirect effects on psychotic experiences via affective disturbance or, *vice versa*.

**Conclusions:**

Our findings tentatively suggest a rapid vicious cycle of stress impacting psychotic experiences via affective disturbances, which does, however, not seem to be consistently modified by familial liability to psychosis.

## Introduction

Recently, the psychosis phenotype has been widely characterized by psychotic experiences that are temporally and phenomenologically continuous with psychotic disorder. Supporting the notion of this extended psychosis phenotype, subclinical expressions of psychotic symptoms are prevalent in the general population (Linscott & van Os, 2013) and associated with an increased risk for developing a psychotic disorder (Fusar-Poli et al., 2013; Linscott & van Os, 2013). There is further evidence that subclinical psychotic experiences are associated with a family history of psychotic disorder (McGrath et al., 2015), suggesting this extended phenotype reflects in part the distributed familial psychosis liability, which is often used as a proxy for genetic risk (although confounded by socio-environmental factors) (van Os, Rutten, & Poulton, 2008). In recent years, studies have implicated a variety of different putative, psychological mechanisms that may be involved in the development and persistence of psychotic experiences in individuals with increased liability to psychosis (EU-GEI, 2014; Freeman & Garety, 2014; Garety, Bebbington, Fowler, Freeman, & Kuipers, 2007; Howes & Murray, 2014; Murray, 2017; van Os, Kenis, & Rutten, 2010; van Os et al., 2008).

Elevated stress sensitivity is a psychological mechanism that has been widely studied in daily life using the Experience Sampling Method (ESM; Myin-Germeys & van Os, 2007; Myin-Germeys et al., 2009, 2018; Oorschot, Kwapil, Delespaul, & Myin-Germeys, 2009). Stress sensitivity has been conceptualized as increased negative affect and psychotic experiences in response to minor stressors in daily life and has been found in both individuals with an increased familial and psychometric risk for psychosis as well as individuals diagnosed with a psychotic disorder (Collip et al., 2011; Lataster et al., 2009; Myin-Germeys & van Os, 2007; Myin-Germeys, van Os, Schwartz, Stone, & Delespaul, 2001; Palmier-Claus, Dunn, & Lewis, 2012; Reininghaus et al., 2016b, 2016c; van der Steen et al., 2017). Also, several models propose that the effects of stress on psychotic experiences are partly mediated through experiences of affective disturbance (Garety et al., 2007; Myin-Germeys & van Os, 2007). Elevated emotional reactivity to minor stress was associated with more intense psychotic experiences in daily life in a group of patients with a first episode of psychosis when compared to healthy controls (Reininghaus et al., 2016c; van der Steen et al., 2017). Also, independently of stress, affective disturbance has been associated with psychotic experiences across different levels of psychosis liability (Bentall et al., 2009; Fowler et al., 2012; Kramer et al., 2014; Thewissen et al., 2011; Varghese et al., 2011). Elevated levels of negative affect, for instance, have been found to precede the experiences of paranoia in individuals with psychotic disorder, in individuals with increased psychometric risk (Thewissen et al., 2011) as well as in a general population twin sample (Lataster et al., 2009).

However, to date, little data have been published on the reverse of the above suggested pathway. Psychotic experiences themselves may be seen as a source of distress and are commonly linked to disturbances in affect (Kelleher et al., 2015; Klippel et al., 2017b; van der Steen et al., 2017). Affective disturbance may then be driving the appraisal of daily events, experiences and contexts as stressful. Psychotic experiences may, therefore, as well be seen as preceding, rather than being merely a *consequence* of, momentary stress. In line with this, recent work by Rapado-Castro, McGorry, Yung, Calvo, and Nelson (2015) suggests a link between subclinical psychotic experiences and distress in at-risk individuals. This, in turn, has been found to be associated with an increased risk of transition to psychosis (Kramer et al., 2014).

Although the body of literature on the link between minor daily stress, affective disturbances and psychotic experiences is growing, and several integrated models have been proposed (Howes & Murray, 2014; Morgan, Charalambides, Hutchinson, & Murray, 2010), to date, only little attention has been paid to how these processes combine in the formation of psychotic experiences in daily life. Using cross-sectional multilevel mediation models, a recent study by our group showed that minor daily stress increases psychotic experiences via pathways through affective disturbances (Klippel et al., 2017a). This indirect effect was greater in individuals with an at-risk mental state and individuals with a first-episode psychosis than in healthy control subjects. In another recent study, we applied the network approach to psychopathology to elucidate the dynamic interplay of momentary experiences, contextual factors and psychotic experiences longitudinally (Klippel et al., 2017b). Findings implied that affective disturbance had an intermediary position between minor daily stress and psychotic experiences.

The aim of the current study was twofold. First, we sought to investigate cross-sectionally how momentary stress and affective disturbance combine to increase the intensity of psychotic experiences in daily life, and *vice versa*, thereby aiming to replicate our previous findings (Klippel et al., 2017a) in another population of individuals with increased familial liability to psychosis. Second, we attempted to test these pathways longitudinally, applying multilevel cross-lagged panel models. We used the ESM in three groups varying in their familial liability to psychosis: individuals with psychotic disorder, first-degree relatives of individuals with psychotic disorder and healthy control individuals. Specifically, the current study tested the following main hypotheses: in all three groups, (i) the cross-sectional effect of momentary stress on psychotic experiences is mediated by affective disturbance; (ii) the cross-sectional effect of psychotic experiences on momentary stress is mediated by affective disturbance; (iii) the longitudinal effect of momentary stress on psychotic experiences is mediated by affective disturbance; and (iv) the longitudinal effect of psychotic experiences on momentary stress is mediated by affective disturbance. We further hypothesized that all these indirect effects are greater in (a) individuals with psychotic disorder than in controls, (b) relatives than in controls and (c) individuals with psychotic disorder than in relatives.

## Methods

### Samples

We used data from six different studies (Collip et al., 2011; Lataster et al., 2011a, 2011b; Myin-Germeys et al., 2001; Thewissen, Bentall, Lecomte, van Os, & Myin-Germeys, 2008; van der Steen et al., 2017) included in the pooled Maastricht MERGE database (release number 4.5; see online Supplementary Table S1 for in- and exclusion criteria of these studies) that all used a similar ESM protocol. Participants were classified either as (i) ‘healthy’ control individuals (i.e., neither a personal diagnosis nor a family history of psychotic disorder/symptoms), (ii) first-degree relatives of individuals with a psychotic disorder or (iii) individuals with a psychotic disorder.

All studies included in this paper were approved by the local medical ethics committee. All further procedures and analyses were performed according to the ethical standards formulated by this committee.

### Experience Sampling Method

In all studies, ESM (a structured diary technique) was used to study minor stress in everyday life (see Table 1) (Myin-Germeys et al., 2018). Individuals received a diary and a wristwatch, which was programmed to beep 10 times a day (between 7:30 h and 22:30 h) for 5 [Aripiprazol study (Lataster et al., 2011b)] or 6 days (remaining studies) at semi-random intervals (random within 90 min time frames). Thus, the time lag between the measurements was, on average, approximately 90 min. Further information on the ESM procedure and the variables used in the current study are presented in Table 1.

**Table 1.** ESM procedure^a^ and measures of stress, negative affect and psychotic experiences

Domain	ESM measures
Momentary stress	We used a composite measure of momentary stress combining aspects of event-related stress, activity-related stress and social stress. This composite score was calculated by computing the row mean.
Event-related	Event-related stress was assessed with 1 item. In this item participants rated the most important event since the last beep on a 7-point Likert scale (−3 = ‘very unpleasant’ to 3 = ‘very pleasant’). The item was reverse coded with higher ratings indicating higher levels of stress (a rating of −3 coded as 7 and a rating of 3 coded as 1).
Activity-related	The activity-related stress scale consisted of 3 items (‘This activity is difficult for me’, ‘I would prefer doing something else’, ‘This activity is challenging’) rated on a 7-point Likert (1 = ‘not at all’ to 7 = ‘very much’).
Social	Social stress was measured with a mean of 2 items. First, participants had to answer the question ‘Who am I with?’ (e.g. partner, family, friends, colleagues, acquaintances, strangers, others, nobody). Then, participants were asked to rate their current social context on a 7-point Likert scale (1 = ‘not at all’ to 7 = ‘very much’) with 2 questions: (1) ‘I would prefer to be alone [if with someone]’; (2) ‘I find being with these people pleasant [if with someone]’ (reversed).
Negative affect	We used the mean of five ESM items to measure negative affect. In line with earlier work we used the following items asking participants to rate the extent to which they felt down, lonely, anxious, insecure and annoyed on a 7-point Likert scale (1 = ‘not at all’ to 7 = ‘very much’) (Klippel et al., 2017a, 2017b).
Psychotic experiences	We used the following six items covering different aspects of mental states that have been associated with psychotic experiences: ‘I feel paranoid’, ‘I feel unreal’, ‘I hear things that aren't really there’, ‘I see things that aren't really there’, ‘I can't get these thoughts out of my head’ and ‘I feel like I am losing control’ (Myin-Germeys et al., 2005a, 2005b; Reininghaus et al., 2016a, 2016b; Table 1b, c). Participants were asked to rate the intensity of psychotic experiences on a 7-point Likert scale (1 = ‘not at all’ to 7 = ‘very much’). High internal consistency and good concurrent validity with interviewer-rated measures of psychotic experiences have been previously reported (Myin-Germeys et al., 2001, 2005a, 2005b, 2009; Palmier-Claus et al., 2012, 2011; Reininghaus et al., 2016a, 2016b).

a *ESM procedure*: Over a period of six consecutive days, participants were equipped with a diary and a wristwatch which was programmed to give a signal 10 times a day. Participants were explained to stop their activity and respond to the above items when prompted by the beep signal as part of a comprehensive diary questionnaire assessing activities, feelings, thoughts, behaviours, social situations and surroundings in daily life. The assessment period started on any day of the week as selected by the participant and they were asked to note the time they filled out the ESM questionnaire. Participants also noted the time of the assessment. Reports completed later than 15 min after the signal were excluded from the analysis. In order to maximize the number of observations for every participant, participants were contacted at least once during the assessment period to assess instruction adherence, identify any concerns associated with the method and help participants with any problems in completing the ESM questionnaire. The participants' reactivity to and compliance with the method was assessed in a debriefing session at the end of the assessment period. In order to be included in the analysis, participants had to provide valid responses to at least one-third of the beep signals.

### Statistical analysis

Data from ESM studies have a hierarchical structure with multiple observations nested within subjects. We therefore fitted multilevel moderated mediation models in Mplus, Version 7 (Muthén & Muthèn, 1998–2017), to control for within-subject clustering of multiple observations. We did this using the MLR and MLF estimators, which allowed us to use all available data under the relatively unrestrictive assumption that data are missing at random if all variables associated with missing values are included in the model (Preacher, 2015; Preacher, Rucker, & Hayes, 2007). We used a two-level model, where multiple observations (level-1) were treated as nested within subjects (level-2).

#### Cross-sectional multilevel moderated mediation models

**Fig. 1.**
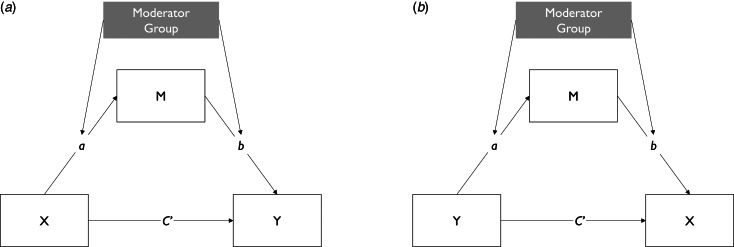
Schematic display of cross-sectional moderated mediation models. Display of pathways tested within each group: (*a*) momentary stress (*X*) on psychotic experiences (*Y*) via negative affect (*M*); (2) psychotic experiences (*Y*) on momentary stress (*X*) via negative affect (*M*). All models were controlled for age and gender.

The total effect of momentary stress in daily life (level-1) on the intensity of psychotic experiences (level-1) was apportioned into direct and indirect effects through negative affect using the product of coefficients strategy. With this strategy, we can quantify the point estimate of the indirect effect as the product of the coefficient of the independent variable on the mediator variable (path a) and the coefficient of mediator variable on the dependent variable (path b). Given its advantages over other methods in the context of multilevel mediation models, we used an R package by Selig and Preacher for computing Monte Carlo confidence intervals and assessing the statistical significance of indirect effects (Preacher & Selig, 2012; Preacher et al., 2007). Group (patients, relatives, controls) was used as the moderator variable (level-2) of direct and conditional indirect effects in all analyses. We did this based on a multilevel moderated mediation approach, where the moderator variable is the predictor of the a and b paths (see Fig. 1) and the strength of the indirect effect of the level-1 independent variable depends on the level-2 moderator variable (Bauer, Preacher, & Gil, 2006; Preacher et al., 2007). By doing this, we could test whether conditional indirect effects were greater in (a) patients than in controls, (b) relatives than in controls and (c) patients than in relatives by computing differences in conditional indirect effects using the model constraint command in Mplus (Muthén & Muthèn, 1998–2017) and calculating respective Monte Carlo confidence intervals (Bauer et al., 2006; Preacher & Selig, 2012; Preacher et al., 2007). Further, we calculated the proportion mediated, a widely used measure of effect size in mediation literature (MacKinnon, Fairchild, & Fritz, 2007; Shrout & Bolger, 2002), as the ratio of the indirect effect to the total effect.

#### Longitudinal multilevel moderated mediation models

**Fig. 2.**
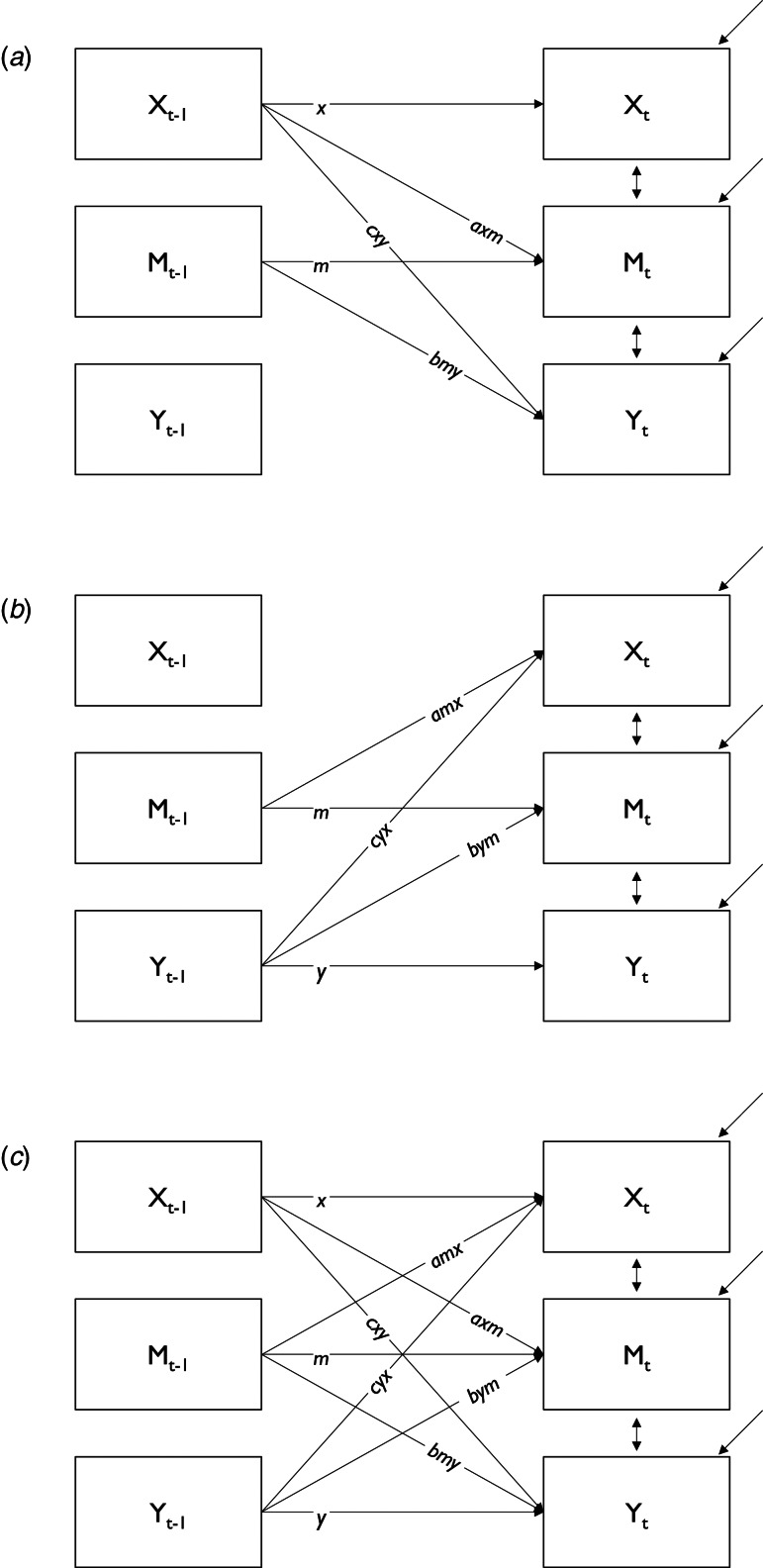
Schematic display of cross-lagged panel models. Display of pathways tested within each group: (*a*) momentary stress at *t*−1 (*X_t_*_−1_) on psychotic experiences at *t* (*Y_t_*) via negative affect at *t* (*M_t_*); (*b*) psychotic experiences at *t*−1 (*Y_t_*_−1_) on momentary stress at *t* (*X_t_*) via negative affect at *t* (*M_t_*); (*c*) all pathways tested in one comprehensive model. All models controlled for age and gender.

In a first step, we fitted an autoregressive model to estimate autoregressive effects of momentary stress at *t*−1 on momentary stress at *t*, negative affect at *t*−1 on negative affect at *t* and psychotic experiences at *t*−1 on psychotic experiences at *t*. In order to assess longitudinal mediation, we fitted a multilevel moderated cross-lagged panel model for a half-longitudinal design (CLPM) as proposed by Preacher (2015) and estimated all covariances between random intercepts and random slopes. In a half-longitudinal design, an indirect effect of an independent variable (*X*) on a dependent variable (*Y*) via a mediator variable (*M*) is estimated using data of two measurement occasions (see Fig. 2). The total effect of momentary stress at *t*−1 on psychotic experiences at *t* was apportioned into direct and indirect effects of negative affect at *t*, again, using the product of coefficients strategy. Using this strategy, we can quantify the point estimate of the indirect effect as the product of the coefficient of the independent variable on the mediator variable (path a) and the coefficient of mediator variable on the dependent variable (path b). For example, the point estimate of the indirect effect of momentary stress (*X_t_*_−1_) on psychotic experiences (*Y_t_*) through negative affect (*M_t_*) is quantified as the product of the coefficient of momentary stress (*X_t_*_−1_) on negative affect (*M_t_*) (path *axm*, in Fig. 2*c*) and the coefficient of negative affect (*M_t_*_−1_) on psychotic experiences (*Y_t_*) (path *bmy*, in Fig. 2*c*). In the same model, we proceeded likewise with the effect of psychotic experiences at *t*−1 on momentary stress at *t* via negative affect at *t*. Monte Carlo confidence intervals were computed for indirect effects according to the above-mentioned procedure (Preacher & Selig, 2012; Preacher et al., 2007). Group (patients, relatives, controls) was used as the moderator variable (level-2) of direct and conditional indirect effects in the analyses. Differences in conditional indirect effects between groups were subsequently computed using the model constraint command in Mplus (Muthén & Muthèn, 1998–2017). Prior to running this comprehensive model, we fitted two separate models: one with pathways from momentary stress to psychotic experiences through negative affect (Fig. 2*a*) and another one including pathways from psychotic experiences to momentary stress via negative affect (Fig. 2*b*).

## Results

### Basic sample characteristics

Basic sample characteristics and aggregate ESM scores for momentary stress, negative affect and psychotic experiences are presented in Table 2. Both patients and relatives differed significantly from controls in aggregate ESM scores for momentary stress, negative affect and psychotic experiences. Interestingly, aggregate momentary stress scores of relatives were similar to those of patients, whereas their aggregate scores of negative affect and psychotic experiences were more similar to controls.

**Table 2.** Sample characteristics and aggregate ESM scores for momentary stress, negative affect and psychotic experiences in patients, relatives and controls

	Patients	Relatives	Controls	Test statistic	*p*
N	245	165	244		
Age (years), mean (s.d.)	35.3 (10.8)	36.7 (12.7)	36.4 (12.4)	*F* = 37.96, df = 2	<0.001
Gender, *n* (%)				Χ^2^ = 0.51, df = 2	0.77
Male	111 (46)	68 (41)	111 (44)		
Female	132 (54)	97 (59)	132 (56)		
Mean ESM scores (s.d.)					
Momentary stress	2.68 (1.03)	2.60 (0.95)	2.49 (0.96)		
Negative affect	1.89 (1.09)	1.34 (0 0.67)	1.29 (0.55)		
Psychotic experiences	1.64 (0.93)	1.11 (0.31)	1.09 (0.25)		

	Patients *v*. controls		Patients *v*. relatives		Relatives *v*. controls	
	*t*	*p*	*t*	*p*	*t*	*p*
Age	−1.18	<0.001	−1.42	<0.001	0.24	0.5
	adj. *B* (95% CI)	*p*	adj. *B* (95% CI)	*p*	adj. *B* (95% CI)	*p*
Momentary stress	−0.193 (−0.282 to −0.104)	<0.001	−0.073 (−0.172 to 0.026)	0.15	−0.120 (−0.219 to −0.021)	0.02
Negative affect	−0.613 (−0.724 to −0.502)	<0.001	−0.559 (−0.682 to −0.436)	<0.001	−0.053 (−0.177 to 0.070)	0.40
Psychotic experiences	−0.560 (−0.654 to −0.466)	<0.001	−0.542 (−0.646 to −0.437)	<0.001	−0.018 (−0.123 to 0.087)	0.74

s.d., standard deviation; *v*., versus; CI, confidence interval.

### Cross-sectional multilevel moderated mediation models

To examine pathways from momentary stress to psychotic experiences via negative affect and the reverse from psychotic experiences to momentary stress via negative affect, we fitted two separate multilevel moderated mediation models (Table 3). The indirect effect of momentary stress on the intensity of psychotic experiences via negative affect was statistically significant at conventional levels (*p* < 0.05) in all groups. This indicated that an increase in stress was associated with higher levels of negative affect, which, in turn, was associated with more intense psychotic experiences. The relative contribution of this indirect effect was larger than the contribution of the direct pathway from momentary stress to psychotic experiences, as indicated by the proportion mediated, which showed that the indirect effect of negative affect accounted for 69–86% of the total effect in patients and controls [*P_M_* exceeds 1.0 in relatives as direct effect is opposite in sign to the indirect effect (Shrout & Bolger, 2002)]. Further, this indirect effect was significantly greater in patients than in controls [adj. *B* = 0.001; 95% CI (0.000–0.002)], weaker in relatives than in controls [adj. *B* = −0.014; 95% CI (−0.016 to −0.012)] and greater in patients than in relatives [adj. *B* = 0.015; 95% CI (0.013–0.017)].

**Table 3.** Total, direct and conditional indirect effects of cross-sectional multilevel moderated mediation models of stress, negative affect and psychotic experiences, *vice versa*^a^

	Patients		Relatives		Controls	
	adj. *B* (95% CI)	*P_M_*	adj. *B* (95% CI)	*P_M_*	adj. *B* (95% CI)	*P_M_*
Momentary stress, negative affect, psychotic experiences						
Direct effect (momentary stress→psychotic experiences)	0.026 (0.022–0.029)		−0.010 (−0.016 to −0.004)		0.009 (0.005–0.012)	
Indirect effect^b^ (momentary stress→negative affect→psychotic experiences)	0.058 (0.057–0.060)	0.69	0.043 (0.041–0.045)	1.30^c^	0.057 (0.056–0.059)	0.86
Total effect	0.084 (0.079–0.089)		0.033 (0.025–0.041)		0.066 (0.061–0.071)	
Momentary psychotic experiences, negative affect, momentary stress						
Direct effect (psychotic experiences→momentary stress)	0.128 (0.107–0.148)		0.141 (0.119–0.162)		0.207 (0.187–0.226)	
Indirect effect^b^ (psychotic experiences→negative affect→momentary stress)	0.208 (0.198–0.218)	0.62	0.323 (0.309–0.337)	0.70	0.355 (0.344–0.366)	0.63
Total effect	0.336 (0.305–0.366)		0.464 (0.428–0.499)		0.562 (0.531–0.592)	

	Patients *v*. controls	Patients *v*. relatives	Relatives *v*. controls
	adj. *B* (95% CI)	adj. *B* (95% CI)	adj. *B* (95% CI)
^b^Δ Indirect effects			
Momentary stress→negative affect→psychotic experiences	0.001 (0.000–0.002)	0.015 (0.013–0.017)	−0.014 (−0.016 to −0.012)
Psychotic experiences→negative affect→momentary stress	−0.147 (−0.151 to −0.143)	−0.115 (−0.126 to −0.104)	−0.032 (−0.043 to −0.021)

s.d., standard deviation; *v*., versus; CI, confidence interval; *P_M_*, proportion mediated.

a Adjusted for age and gender.

c Exceeds 1.0 in case of suppression effects [i.e., if direct effect is opposite in sign to the indirect effect (Shrout & Bolger, 2002)].

Turning to the findings of psychotic experiences on momentary stress, there was evidence that the effect of psychotic experiences on momentary stress was significantly mediated by levels of negative affect in all three groups. Overall, the magnitude of these indirect effects was greater than those of reverse pathways (i.e., effects of stress on psychotic experiences via negative affect). The indirect effect was greatest in controls [adj. *B* = 0.355; 95% CI (0.344–0.366)], followed by relatives [adj. *B* = 0.323; 95% CI (0.309–0.337)] and then patients [adj. *B* = 0.208; 95% CI (0.198–0.218)]. Again, the relative contribution of this indirect effect was greater than the contribution of the direct effect from psychotic experiences to momentary stress, as this indirect effect accounted for 62–70% of the total effect. The indirect effect of psychotic experiences on momentary stress via negative affect followed a gradient across the three groups, i.e., it was weaker in patients than in controls [adj. *B* = −0.147; 95% CI (−0.151 to −0.143)], weaker in relatives than in controls [adj. *B* = −0.032; 95% CI (−0.043 to −0.021)] and weaker in patients than in relatives [adj. *B* = −0.115; 95% CI (−0.216 to −0.104)].

### Longitudinal multilevel moderated mediation models

Model fit statistics are presented in online Supplementary Table S2. The comprehensive cross-lagged panel model showed a significantly better fit to the data than the autoregressive model or separate cross-lagged panel models. Results of the autoregressive model can be found in Table 4. Levels of momentary stress, negative affect and psychotic experiences at *t*−1 were significantly associated with levels at *t* (all *p* = 0.000).

**Table 4.** Autoregressive effects (*t*−1→*t*) of momentary stress, negative affect and psychotic experiences in patients, relatives and controls

	Patients	Relatives	Controls
	*B* (95% CI)	*B* (95% CI)	*B* (95% CI)
Stress*_t_*_−1_→stress*_t_*	0.171 (0.139–0.202)	0.153 (0.120–0.187)	0.146 (0.120–0.171)
Negative affect*_t_*_−1_→negative affect*_t_*	0.286 (0.248–0.325)	0.200 (0.151–0.249)	0.211 (0.174–0.248)
Psychotic experiences*_t_*_−1_→psychotic experiences*_t_*	0.299 (0.251–0.348)	0.249 (0.180–0.318)	0.189 (0.138–0.240)

When we examined the indirect effects of momentary stress on psychotic experiences, and *vice versa*, the indirect effects of psychotic experiences on momentary stress simultaneously in one longitudinal cross-lagged panel model with two time points, there was no evidence that the effect of momentary stress on psychotic experiences was mediated by negative affect in any of the three groups (Table 5).

**Table 5.** Total, direct and conditional indirect effects of longitudinal multilevel moderated mediation models of stress, negative affect and psychotic experiences^a^

	Patients	Relatives	Controls
	adj. *B* (95% CI)	adj. *B* (95% CI)	adj. *B* (95% CI)
Momentary stress, negative affect, psychotic experiences			
Direct effect [momentary stress (*t*−1)→psychotic experiences (*t*)]	−0.007 (−0.022 to 0.007)	0.000 (−0.005 to 0.006)	0.002 (−0.004 to 0.007)
Indirect effect^b^ [Momentary stress (*t*−1)→negative affect (*t*)→psychotic experiences (*t*)]	0.000 (−0.001 to 0.001)	0.001 (−0.001 to 0.001)	0.000 (−0.000 to 0.000)
Total effect	−0.007 (−0.022 to 0.008)	0.001 (−0.005 to 0.007)	0.002 (−0.004 to 0.007)
Psychotic experiences, negative affect, momentary stress			
Direct effect [psychotic experiences (*t*−1)→momentary stress (*t*)]	0.057 (−0.015 to 0.129)	0.034 (−0.092 to 0.159)	0.026 (−0.079 to 0.131)
Indirect effect^b^ [psychotic experiences (*t*−1)→negative affect (*t*)→ momentary stress (*t*)]	0.005 (−0.001 to 0.011)	0.012 (−0.001 to 0.025)	0.006 (−0.001 to 0.013)
Total effect	0.062 (−0.008 to 0.133)	0.045 (−0.080 to 0.170)	0.032 (−0.073 to 0.137)

	Patients *v*. controls	Patients *v*. relatives	Relatives *v*. controls
	adj. *B* (95% CI)	adj. *B* (95% CI)	adj. *B* (95% CI)
Δ Indirect effects			
Momentary stress (*t*−1)→negative affect (*t*)→psychotic experiences (*t*)	0.000 (−0.001 to 0.001)	0.000 (−0.001 to 0.001)	0.001 (0.000–0.001)
Psychotic experiences (*t*−1)→negative affect (*t*)→momentary stress (*t*)	0.001 (−0.010 to 0.008)	−0.007(−0.020 to 0.008)	0.006 (−0.009 to 0.020)

s.d., standard deviation; *v*., versus; CI, confidence interval.

a Adjusted for age and gender.

## Discussion

### Principal findings

It was the central aim of the current study to investigate how stress and affective disturbances combine to increase the intensity of psychotic experiences and to establish a temporal order thereof. We found that, cross-sectionally, an increase in stress was associated with higher levels of negative affect, which, in turn, was associated with more intense psychotic experiences consistently across the three groups. There was evidence of greater indirect effects of momentary stress on psychotic experiences in patients than both relatives and controls, with the weakest effects being evident in relatives. Cross-sectional modelling of data further indicated that the effects of psychotic experiences on momentary stress were mediated by the levels of negative affect in all three groups. The strength of this indirect effect differed significantly in all three groups, with the strongest effects being evident in controls, closely followed by relatives and, then, albeit weaker, patients. Hence, there was no consistent evidence from cross-sectional models that indirect effects increased as liability to psychosis increased. Longitudinal modelling of data, however, provided no evidence of temporal priority of stress and affective disturbances over psychotic experiences, or, *vice versa*, an indirect effect of psychotic experiences on stress by affective disturbances.

### Methodological considerations

The current findings should be viewed in the light of potential limitations. First, longitudinal models in the current study did not yield evidence for longitudinal effects across two measurement occasions. As has been proposed by Shiffman, Stone and Hufford, in ESM research, it is important that assessment schemes fit the phenomenon of interest and the estimation of how rapidly it is expected to vary (Shiffman, Stone, & Hufford, 2008). In our study, lags between measurements were on average 90 min. Possibly, the effects of stress on psychotic experiences, and *vice versa*, may have been too transient to be lingering from one moment to the next. Another possibility is, that lag duration in the current study was too long to detect changes, that, in fact, may be there. One study by Vaessen et al. (2019) showed that particularly patients in the early stages of psychosis may take longer to recover from stress. It would therefore be of interest to investigate whether reducing or increasing the duration of lags (i.e., the time between measurement occasions) would produce different findings. Future studies may further investigate the temporal interplay between stress, affective disturbances and psychotic experiences by systematically manipulating the time that passes between assessment points (Reininghaus, Depp, & Myin-Germeys, 2016a). The results by Vaessen et al. (2019) further beg the question whether findings from the current study would replicate in individuals with early psychosis.

Second, the magnitude of indirect effects of negative affect was comparable to previous studies (Klippel et al., 2017a) and suggests evidence of partial mediation. This implies that there may be other unmeasured factors that are relevant in the pathways to psychosis.

Third, in the current study, we employed cross-sectional models as well as cross-lagged panel models of two measurement occasions to investigate how momentary stress and negative affect combine to increase psychotic experiences, and *vice versa*. Although fitting full cross-lagged panel models of three measurement occasions as described by Preacher (2015) would have been a natural next step, we deem it unlikely that these models would have yielded evidence on temporal order given there was no evidence on this in cross-lagged panel models of two measurement occasions and the magnitude of indirect effects was very small and, for some, even trivial. However, this may be an important extension in the modelling strategy for future research.

Fourth, despite a number of benefits, pooling data from six different ESM studies may possibly entail disadvantages and may have produced a certain heterogeneity within the three groups of our sample. However, study protocols, in- and exclusion criteria were reviewed carefully before combining the six datasets. All studies employed comparable ESM protocols, using watches and booklets, on six (in one study five) consecutive days. Also, the in- and exclusion criteria for patients, relatives and controls (see also online Supplementary Table S1) were comparable across the combined studies. We therefore believe that the heterogeneity has been kept to a minimum in the current study and may not provide a problem.

Fifth, ESM measures are based on subjective reports of participants and may therefore be less reliable, since for example, not all subjects may interpret questions in the same way. In addition, ESM data collection can be very time-intensive and possibly be associated with assessment burden. Previous research, however, has shown that the ESM is a feasible, reliable and valid assessment method in a variety of different populations (Myin-Germeys et al., 2001, 2009, 2018; Palmier-Claus et al., 2012; Rauschenberg, van Os, Goedhart, Schieveld, & Reininghaus, 2020; Reininghaus et al., 2016c). Also, in all of the combined six studies, participants were extensively briefed on the ESM by a trained researcher prior to start of data collection, to ensure correct interpretation of the employed items and proper use of the data booklet and preprogrammed watch (Collip et al., 2011; Lataster et al., 2011b; Lataster, Valmaggia, Lardinois, van Os, & Myin-Germeys, 2013; Myin-Germeys et al., 2001; Thewissen et al., 2008; van der Steen et al., 2017).

### Comparison with previous research

In recent years, elevated reactivity to momentary stress has been suggested to reflect an important putative underlying mechanism in psychotic disorders (Myin-Germeys & van Os, 2007; Myin-Germeys et al., 2001; Palmier-Claus et al., 2012; Reininghaus et al., 2016c). In line with this, individuals with an increased risk for psychosis have been found to experience elevated levels of reactivity to minor stressors in daily life (Collip et al., 2011; Devylder et al., 2013; Myin-Germeys et al., 2001; Palmier-Claus et al., 2012). This has previously been coined the affective pathway to psychosis (Kramer et al., 2014; Myin-Germeys & van Os, 2007). When turning to stress in more general terms, different models of psychosis have posited that the effects of stress are mediated by affective disturbance (Garety et al., 2007; Howes & Murray, 2014; Morgan et al., 2010). A recent study by our group provided new evidence for this proposition and found that the cross-sectional effects of momentary stress on psychotic experiences were indeed mediated by affective disturbances in daily life across different stages along the psychosis continuum (Klippel et al., 2017a). Our findings from cross-sectional models replicate these earlier findings suggesting that the effect of stress on psychotic experiences is mediated through affective disturbance, but, in contrast to what we hypothesized, there was no evidence that this indirect effect increased as familial liability to psychosis increased. What is more, in the reverse model, there was a gradual increase of the indirect effect of psychotic experiences on momentary stress via affective disturbance as familial liability *decreased*, with patients showing the weakest indirect effect. This may in part reflect the effects of illness chronicity or long-term exposure to antipsychotic medication on how strongly psychotic experiences impact the appraisal of events, activities and social situations as stressful via low mood. Finally, there was no evidence from longitudinal cross-lagged panel models that the indirect effects of momentary stress on psychotic experiences were mediated via affective disturbance.

It has been proposed that psychotic experiences themselves may be distressing (Kelleher et al., 2015; van der Steen et al., 2017; Wigman et al., 2011; Yung et al., 2006), and in many cases, it is the experience of distress with symptoms that leads individuals to contact mental health services (Freeman & Garety, 2003). In the current study, we investigated whether psychotic experiences are associated with affective disturbance, which in turn are linked to increases in the experiences of momentary stress. To our knowledge, so far, there is no study that examined this pathway in its entirety in daily life. We found that the effects of psychotic experiences on momentary stress were mediated by negative affect, but these indirect effects did not increase as psychosis liability increased, with patients and, in fact, controls showing the largest effects. Interestingly, the magnitude of indirect effects in this cross-sectional pathway was considerably larger than those of the reverse pathway (from momentary stress to psychotic experiences via affective disturbance). This may tentatively suggest a greater impact of psychotic experiences on stress via negative affect than of stress on psychotic experiences through negative affect. Based on our findings, we can hypothesize that the occurrence of psychotic experiences may alter the appraisal of stress in daily life via experiences of affective disturbance. We believe that this pathway should receive more attention in future ESM studies in order to improve our understanding of the momentary impact that psychotic experiences may have on the individual.

The present work also aimed to investigate whether momentary stress takes temporal priority in exerting its indirect effects on psychotic experiences via affective disturbances to test recently proposed affective pathways to psychosis. As we did not find evidence on indirect temporal effects of momentary stress on psychotic experiences via affective disturbances, or *vice versa*, these findings suggest that, consistent with Kramer et al. (2014), the temporal interplay of stress, affective disturbance and psychotic experiences may be more complex still than was hypothesized and modelled in the current study. Hence, the role of mediating and synergistic effects (and, in fact, mediated synergy) needs to be investigated jointly with other relevant aetiological factors as an important next step. Investigating temporal interplay in daily life is important as a basis for real-time and real-world interventions, such as Ecological Momentary Interventions (EMI; Heron & Smyth, 2010; Myin-Germeys, Klippel, Steinhart, & Reininghaus, 2016; Myin-Germeys et al., 2018; Reininghaus, 2018). Relatives of patients with a psychotic disorder have an increased risk for developing the disorder themselves (Kendler & Diehl, 1993) and have been reported to show increases in the intensity of subtle psychotic experiences and affective disturbance in response to momentary stress (Myin-Germeys, Delespaul, & van Os, 2005a; Myin-Germeys, Marcelis, Krabbendam, Delespaul, & van Os, 2005b). The findings of the current study may, however, point towards a certain resilience in relatives of patients. Both relatives and patients showed similar aggregate levels of momentary stress that were higher than those experienced by controls. However, when looking at aggregate levels of negative affect and psychotic experiences, these were similar in relatives and controls and significantly lower than those of patients. Furthermore, relatives showed the smallest magnitude of indirect effects when compared to the other two groups. Based on these findings, we may speculate that, although relatives experience levels of momentary stress similar to those of patients in everyday life, these are linked to a smaller increase in negative affect and psychotic experiences. Our findings do not support the hypothesis that familial liability modifies how stress impacts psychotic experiences via affective disturbance, which has been proposed previously (Lataster, Collip, Lardinois, van Os, & Myin-Germeys, 2010; Myin-Germeys & van Os, 2007; Myin-Germeys et al., 2001).

## Conclusion

Taken together, we found no evidence to support the temporal priority of momentary stress over affective disturbance and psychotic experiences, *vice versa*. However, findings from cross-sectional models may tentatively suggest a rapid vicious cycle of stress impacting psychotic experiences, and *vice versa*, via affective disturbances. This, in turn, highlights the importance of investigating reciprocal effects between these aspects in future studies. The question, then, remains, whether more rapid cycling of stress, affective disturbances and psychotic experiences may contribute to the persistence of psychotic experiences over time. This would, in turn, open new avenues for identifying and targeting the dynamics of these basic psychological dimensions in daily life and allow for clinical translational research using novel, personalized EMI (Myin-Germeys et al., 2016; Reininghaus et al., 2016a) for targeting these dynamics in the early stages of developing psychotic experiences to prevent their transformation into full-blown psychotic symptoms.

## Supplementary material

For supplementary material accompanying this paper visit https://doi.org/10.1017/S0033291720004894. click here to view supplementary material

## References

[ref1] Bauer, D. J., Preacher, K. J., & Gil, K. M. (2006). Conceptualizing and testing random indirect effects and moderated mediation in multilevel models: New procedures and recommendations. Psychological Methods, 11, 142–163. doi: 10.1037/1082-989X.11.2.142. 16784335

[ref2] Bentall, R. P., Rowse, G., Shryane, N., Kinderman, P., Howard, R., Blackwood, N., … Corcoran, R. (2009). The cognitive and affective structure of paranoid delusions: A transdiagnostic investigation of patients with schizophrenia spectrum disorders and depression. Archives of General Psychiatry, 66, 236–247. doi: 10.1001/archgenpsychiatry.2009.1. 19255373

[ref3] Collip, D., Nicolson, N. A., Lardinois, M., Lataster, T., van Os, J., & Myin-Germeys, I., & G.R.O.U.P (2011). Daily cortisol, stress reactivity and psychotic experiences in individuals at above average genetic risk for psychosis. Psychological Medicine, 41, 2305–2315. doi: 10.1017/S0033291711000602. 21733219

[ref4] Devylder, J. E., Ben-David, S., Schobel, S. A., Kimhy, D., Malaspina, D., & Corcoran, C. M. (2013). Temporal association of stress sensitivity and symptoms in individuals at clinical high risk for psychosis. Psychological Medicine, 43, 259–268. doi: 10.1017/S0033291712001262. 22651857 PMC3716006

[ref5] EU-GEI, European Network of National Network studying Gene-Environment Interactions in Schizophrenia (2014). Identifying gene-environment interactions in schizophrenia: Contemporary challenges for integrated, large-scale investigations. Schizophrenia Bulletin, 40, 729–736. doi: 10.1093/schbul/sbu069. 24860087 PMC4059449

[ref6] Fowler, D., Hodgekins, J., Garety, P., Freeman, D., Kuipers, E., Dunn, G., … Bebbington, P. E. (2012). Negative cognition, depressed mood, and paranoia: A longitudinal pathway analysis using structural equation modeling. Schizophrenia Bulletin, 38, 1063–1073. doi: 10.1093/schbul/sbr019. 21474550 PMC3446231

[ref7] Freeman, D., & Garety, P. A. (2003). Connecting neurosis and psychosis: The direct influence of emotion on delusions and hallucinations. Behavioral Research & Therapy, 41, 923–947. doi: 10.1016/S0005-7967(02)00104-3. 12880647

[ref8] Freeman, D., & Garety, P. (2014). Advances in understanding and treating persecutory delusions: A review. Social Psychiatry and Psychiatric Epidemiology, 49, 1179–1189. doi: 10.1007/s00127-014-0928-7. 25005465 PMC4108844

[ref9] Fusar-Poli, P., Borgwardt, S., Bechdolf, A., Addington, J., Riecher-Rössler, A., Schultze-Lutter, F., … Yung, A.(2013). The psychosis high-risk state: A comprehensive state-of-the-art review. JAMA Psychiatry, 70, 107–120. doi: 10.1001/jamapsychiatry.2013.269. 23165428 PMC4356506

[ref10] Garety, P. A., Bebbington, P., Fowler, D., Freeman, D., & Kuipers, E. (2007). Implications for neurobiological research of cognitive models of psychosis: A theoretical paper. Psychological Medicine, 37, 1377–1391. doi: 10.1017/S003329170700013X. 17335638

[ref11] Heron, K. E., & Smyth, J. M. (2010). Ecological momentary interventions: Incorporating mobile technology into psychosocial and health behaviour treatments. British Journal of Health Psychology, 15, 1–39. doi: 10.1348/135910709X466063. 19646331 PMC2800172

[ref12] Howes, O. D., & Murray, R. M. (2014). Schizophrenia: An integrated sociodevelopmental-cognitive model. The Lancet, 383, 1677–1687. doi: 10.1016/S0140-6736(13)62036-X. PMC4127444 24315522

[ref13] Kelleher, I., Wigman, J. T. W., Harley, M., O'Hanlon, E., Coughlan, H., Rawdon, C., … Cannon, M. (2015). Psychotic experiences in the population: Association with functioning and mental distress. Schizophrenia Research, 165, 9–14. doi: 10.1016/j.schres.2015.03.020. 25868930

[ref14] Kendler, K. S., & Diehl, S. R. (1993). The genetics of schizophrenia: A current, genetic-epidemiologic perspective. Schizophrenia Bulletin, 19, 261–285. doi: 10.1093/schbul/19.2.261. 8322035

[ref15] Klippel, A., Myin-Germeys, I., Chavez-Baldini, U., Preacher, K. J., Kempton, M., Valmaggia, L., … Reininghaus, U. (2017a). Modeling the interplay between psychological processes and adverse, stressful contexts and experiences in pathways to psychosis: An experience sampling study. Schizophrenia Bulletin, 43, 302–315. doi: 10.1093/schbul/sbw185. 28204708 PMC5605264

[ref16] Klippel, A., Viechtbauer, W., Reininghaus, U., Wigman, J., van Borkulo, C., Myin-Germeys, I., … Wichers, M. (2017b). The cascade of stress: A network approach to explore differential dynamics in populations varying in risk for psychosis. Schizophrenia Bulletin, 44, 328–337. doi: 10.1093/schbul/sbx037. PMC5815145 28338969

[ref17] Kramer, I., Simons, C. J., Wigman, J. T., Collip, D., Jacobs, N., Derom, C., … Wichers, M. (2014). Time-lagged moment-to-moment interplay between negative affect and paranoia: New insights in the affective pathway to psychosis. Schizophrenia Bulletin, 40, 278–286. doi: 10.1093/schbul/sbs194. 23407984 PMC3932075

[ref18] Lataster, J., Collip, D., Ceccarini, J., Haas, D., Booij, L., van Os, J., … Myin-Germeys, I. (2011a). Psychosocial stress is associated with in vivo dopamine release in human ventromedial prefrontal cortex: A positron emission tomography study using [(1)(8)F]fallypride. Neuroimage, 58, 1081–1089. doi: 10.1016/j.neuroimage.2011.07.030. 21801840

[ref19] Lataster, T., Collip, D., Lardinois, M., van Os, J., & Myin-Germeys, I. (2010). Evidence for a familial correlation between increased reactivity to stress and positive psychotic symptoms. Acta Psychiatrica Scandinavica, 122, 395–404. doi: 10.1111/j.1600-0447.2010.01566.x. 20491716

[ref20] Lataster, J., Myin-Germeys, I., Wichers, M., Delespaul, P. A., van Os, J., & Bak, M. (2011b). Psychotic exacerbation and emotional dampening in the daily life of patients with schizophrenia switched to aripiprazole therapy: A collection of standardized case reports. Therapeutic Advances in Psychopharmacology, 1, 145–151. doi: 10.1177/2045125311419552. 23983939 PMC3736906

[ref21] Lataster, T., Valmaggia, L., Lardinois, M., van Os, J., & Myin-Germeys, I. (2013). Increased stress reactivity: A mechanism specifically associated with the positive symptoms of psychotic disorder. Psychological Medicine, 43, 1389–1400. doi: 10.1017/S0033291712002279. 23111055

[ref22] Lataster, T., Wichers, M., Jacobs, N., Mengelers, R., Derom, C., Thiery, E., … Myin-Germeys, I. (2009). Does reactivity to stress cosegregate with subclinical psychosis? A general population twin study. Acta Psychiatrica Scandinavica, 119, 45–53. doi: 10.1111/j.1600-0447.2008.01263.x. 18822092

[ref23] Linscott, R. J., & van Os, J. (2013). An updated and conservative systematic review and meta-analysis of epidemiological evidence on psychotic experiences in children and adults: On the pathway from proneness to persistence to dimensional expression across mental disorders. Psychological Medicine, 43, 1133–1149. doi: 10.1017/S0033291712001626. 22850401

[ref24] MacKinnon, D. P., Fairchild, A. J., & Fritz, M. S. (2007). Mediation analysis. Annual Reviews of Psychology, 58, 593–614. doi: 10.1146/annurev.psych.58.110405.085542. PMC2819368 16968208

[ref25] McGrath, J. J., Saha, S., Al-Hamzawi, A., Alonso, J., Bromet, E. J., Bruffaerts, R., … Kessler, R. C. (2015). Psychotic experiences in the general population: A cross-national analysis based on 31,261 respondents from 18 countries. JAMA Psychiatry, 72, 697–705. doi: 10.1001/jamapsychiatry.2015.0575. 26018466 PMC5120396

[ref26] Morgan, C., Charalambides, M., Hutchinson, G., & Murray, R. M. (2010). Migration, ethnicity, and psychosis: Toward a sociodevelopmental model. Schizophrenia Bulletin, 36, 655–664. doi: 10.1093/schbul/sbq051. 20513653 PMC2894585

[ref27] Murray, R. M. (2017). 30 years on: How the neurodevelopmental hypothesis of schizophrenia morphed into the developmental risk factor model of psychosis. Schizophrenia Bulletin, 43, 1190–1196. doi: 10.1093/schbul/sbx121. 28981842 PMC5737804

[ref28] Muthén, L. K., & Muthèn, B. O. (1998–2017). Mplus Version 8 [statistical software]. Los Angeles, CA.

[ref29] Myin-Germeys, I., Delespaul, P., & van Os, J. (2005a). Behavioural sensitization to daily life stress in psychosis. Psychological Medicine, 35, 733–741. doi: 10.1017/S0033291704004179. 15918350

[ref30] Myin-Germeys, I., Kasanova, Z., Vaessen, T., Vachon, H., Kirtley, O., Viechtbauer, W., & Reininghaus, U. (2018). Experience sampling methodology in mental health research: New insights and technical developments. World Psychiatry, 17, 123–132. doi: 10.1002/wps.20513. 29856567 PMC5980621

[ref31] Myin-Germeys, I., Klippel, A., Steinhart, H., & Reininghaus, U. (2016). Ecological momentary interventions in psychiatry. Current Opinion in Psychiatry, 29, 258–263. doi: 10.1097/YCO.0000000000000255. 27153125

[ref32] Myin-Germeys, I., Marcelis, M., Krabbendam, L., Delespaul, P., & van Os, J. (2005b). Subtle fluctuations in psychotic phenomena as functional states of abnormal dopamine reactivity in individuals at risk. Biological Psychiatry, 58, 105–110. doi: 10.1016/j.biopsych.2005.02.012. 16038680

[ref33] Myin-Germeys, I., Oorschot, M., Collip, D., Lataster, J., Delespaul, P., & van Os, J. (2009). Experience sampling research in psychopathology: Opening the black box of daily life. Psychological Medicine, 39, 1533–1547. doi: 10.1017/S0033291708004947. 19215626

[ref34] Myin-Germeys, I., & van Os, J. (2007). Stress-reactivity in psychosis: Evidence for an affective pathway to psychosis. Clinical Psychology Review, 27, 409–424. doi: 10.1016/j.cpr.2006.09.005. 17222489

[ref35] Myin-Germeys, I., van Os, J., Schwartz, J. E., Stone, A. A., & Delespaul, P. A. (2001). Emotional reactivity to daily life stress in psychosis. Archives of General Psychiatry, 58, 1137–1144. doi: 10.1001/archpsyc.58.12.1137. 11735842

[ref36] Oorschot, M., Kwapil, T., Delespaul, P., & Myin-Germeys, I. (2009). Momentary assessment research in psychosis. Psychological Assessment, 21, 498–505. doi: 10.1037/a0017077. 19947784

[ref37] Palmier-Claus, J. E., Dunn, G., & Lewis, S. W. (2012). Emotional and symptomatic reactivity to stress in individuals at ultra-high risk of developing psychosis. Psychological Medicine, 42, 1003–1012. doi: 10.1146/annurev-psych-010814-015258. 22067414

[ref38] Palmier-Claus, J. E., Myin-Germeys, I., Barkus, E., Bentley, L., Udachina, A., Delespaul, P. A. E. G., … Dunn, G.. (2011). Experience sampling research in individuals with mental illness: reflections and guidance. Acta Psychiatrica Scandinavica, 123(1), 12–20. 10.1111/j.1600-0447.2010.01596.x. 20712828

[ref39] Preacher, K. J. (2015). Advances in mediation analysis: A survey and synthesis of new developments. Annual Review of Psychology, 66, 825–852. doi: 10.1146/annurev-psych-010814-015258. 25148853

[ref40] Preacher, K. J., Rucker, D. D., & Hayes, A. F. (2007). Addressing moderated mediation hypotheses: Theory, methods, and prescriptions. Multivariate Behavioral Research, 42, 185–227. doi: 10.1080/00273170701341316. 26821081

[ref41] Preacher, K. J., & Selig, J. P. (2012). Advantages of Monte Carlo confidence intervals for indirect effects. Communication Methods and Measures, 6, 77–98. doi: 10.1080/19312458.2012.679848.

[ref42] Rapado-Castro, M., McGorry, P. D., Yung, A., Calvo, A., & Nelson, B. (2015). Sources of clinical distress in young people at ultra high risk of psychosis. Schizophrenia Research, 165, 15–21. doi: 10.1016/j.schres.2015.03.022. 25890793

[ref43] Rauschenberg, C., van Os, J., Goedhart, M., Schieveld, J. N. M., & Reininghaus, U. (2020). Bullying victimization and stress sensitivity in help-seeking youth: Findings from an experience sampling study. European Journal of Child and Adolescent Psychiatry. Advance online publication. doi: 10.1007/s00787-020-01540-5. PMC8041697 32405792

[ref44] Reininghaus, U. (2018). [Ecological momentary interventions in psychiatry: The momentum for change in daily social context]. Psychiatrische Praxis, 45, 59–61. doi: 10.1055/s-0044-101986. 29495051

[ref45] Reininghaus, U., Depp, C. A., & Myin-Germeys, I. (2016a). Ecological interventionist causal models in psychosis: Targeting psychological mechanisms in daily life. Schizophrenia Bulletin, 42, 264–269. doi: 10.1093/schbul/sbv193. 26707864 PMC4753613

[ref46] Reininghaus, U., Gayer-Anderson, C., Valmaggia, L., Kempton, M. J., Calem, M., Onyejiaka, A., … Morgan, C. (2016b). Psychological processes underlying the association between childhood trauma and psychosis in daily life: An experience sampling study. Psychological Medicine, 46, 2799–2813. doi: 10.1017/S003329171600146X. 27400863 PMC5358473

[ref47] Reininghaus, U., Kempton, M. J., Valmaggia, L., Craig, T. K., Garety, P., Onyejiaka, A., … Morgan, C. (2016c). Stress sensitivity, aberrant salience, and threat anticipation in early psychosis: An experience sampling study. Schizophrenia Bulletin, 42, 712–722. doi: 10.1093/schbul/sbv190. 26834027 PMC4838104

[ref48] Shiffman, S., Stone, A. A., & Hufford, M. R. (2008). Ecological momentary assessment. Annual Review of Clinical Psychology, 4, 1–32. doi: 10.1146/annurev.clinpsy.3.022806.091415. 18509902

[ref49] Shrout, P. E., & Bolger, N. (2002). Mediation in experimental and nonexperimental studies: New procedures and recommendations. Psychological Methods, 7, 422–445. doi: 10.1037/1082-989X.7.4.422. 12530702

[ref50] Thewissen, V., Bentall, R. P., Lecomte, T., van Os, J., & Myin-Germeys, I. (2008). Fluctuations in self-esteem and paranoia in the context of daily life. Journal of Abnormal Psychology, 117, 143–153. doi: 10.1037/0021-843X.117.1.143. 18266492

[ref51] Thewissen, V., Bentall, R. P., Oorschot, M., à Campo, J., van Lierop, T., van Os, J., & Myin-Germeys, I. (2011). Emotions, self-esteem, and paranoid episodes: An experience sampling study. British Journal of Clinical Psychology, 50, 178–195. doi: 10.1348/014466510X508677. 21545450

[ref52] Vaessen, T., Viechtbauer, W., van der Steen, Y., Gayer-Anderson, C., Kempton, M. J., Valmaggia, L., … Myin-Germeys, I. (2019). Recovery from daily-life stressors in early and chronic psychosis. Schizophrenia Research, 213, 32–39. doi: 10.1016/j.schres.2019.03.011. 30930036

[ref53] van der Steen, Y., Gimpel-Drees, J., Lataster, T., Viechtbauer, W., Simons, C. J. P., Lardinois, M., … Myin-Germeys, I. (2017). Clinical high risk for psychosis: The association between momentary stress, affective and psychotic symptoms. Acta Psychiatrica Scandinavica, 136, 63–73. doi: 10.1111/acps.12714. 28260264

[ref54] van Os, J., Kenis, G., & Rutten, B. P. F. (2010). The environment and schizophrenia. Nature, 468, 203–212. 21068828 10.1038/nature09563

[ref55] van Os, J., Rutten, B. P., & Poulton, R. (2008). Gene-environment interactions in schizophrenia: Review of epidemiological findings and future directions. Schizophrenia Bulletin, 34, 1066–1082. doi: 10.1093/schbul/sbn117. 18791076 PMC2632485

[ref56] Varghese, D., Scott, J., Welham, J., Bor, W., Najman, J., O'Callaghan, M., … McGrath, J. (2011). Psychotic-like experiences in major depression and anxiety disorders: A population-based survey in young adults. Schizophrenia Bulletin, 37, 389–393. doi: 10.1093/schbul/sbp083. 19687152 PMC3044630

[ref57] Wigman, J. T. W., Vollebergh, W. A. M., Raaijmakers, Q. A. W., Iedema, J., van Dorsselaer, S., Ormel, J., … van Os, J. (2011). The structure of the extended psychosis phenotype in early adolescence – a cross-sample replication. Schizophrenia Bulletin, 37, 850–860. doi: 10.1093/schbul/sbp154. 20044595 PMC3122288

[ref58] Yung, A. R., Buckby, J. A., Cotton, S. M., Cosgrave, E. M., Killackey, E. J., Stanford, C., … McGorry, P. D. (2006). Psychotic-like experiences in nonpsychotic help-seekers: Associations with distress, depression, and disability. Schizophrenia Bulletin, 32, 352–359. doi: 10.1093/schbul/sbj018. 16254060 PMC2632224

