# Supplementary material for: Modelling the temporal interplay between stress and affective disturbances in pathways to psychosis: an experience sampling study
Source: Psychol Med. 2021 Mar 8;52(13):2776–85. doi: 10.1017/S0033291720004894 (PMC9647515; doi:10.1017/S0033291720004894)
Supplement: Supplementary file 1 [file S0033291720004894sup001.docx]

**SUPPLEMENTARY MATERIALS**

| **Table S1.** Overview of in- and exclusion criteria of merged studies | | | |
| --- | --- | --- | --- |
| Study | N | |  |
| PREVENT  (van der Steen *et al.*, 2017) | N=26  Healthy controls | | Inclusion criteria controls:   1. age between 18 and 45   Exclusion criteria controls:   1. current axis I disorder as assessed with the Structured Clinical Interview for DSM-IV (Wittchen *et al.*, 2011) 2. family history of psychotic disorder |
| MAPS  (Myin-Germeys *et al.*, 2001) | N=139  Healthy controls (n=49); First-degree relative (n=47);  Psychotic disorder (n=43) | | Inclusion criteria for all participants:   1. age between 18 and 55 2. sufficient command of the Dutch language   Exclusion criteria controls:   1. current use of psychotropic medication 2. family or personal history of psychotic symptoms   Inclusion criteria relatives:   1. first-degree relatives with a lifetime occurrence of psychotic symptoms   Exclusion criteria relatives:   1. lifetime occurrence of psychotic symptoms   Inclusion criteria patients:   1. normal physical examination results 2. lifetime occurrence of psychotic symptoms as assessed with the Life Chart , the Brief Psychiatric Rating Scale, the Positive and Negative Syndrome Scale (PANSS) (Kay *et al.*, 1987), and the Operational Criteria Checklist for Psychotic illness (OPCRIT) (McGuffin *et al.*, 1991)   Exclusion criteria patients:   1. endocrine, cardiovascular, or brain disease 2. excessive use of alcohol (≥5 standard units per day) 3. weekly use of illicit drugs 4. history of head injury with loss of consciousness 5. need for inpatient care |
| GROUP  (Collip *et al.*, 2011) | N=219  Healthy controls (n=83);  First-degree relative (n=70);  Psychotic disorder (n=66) | | Inclusion criteria for all participants:   1. age between 16 and 55 2. sufficient command of the Dutch language   Exclusion criteria controls:   1. first-degree relative with a psychotic disorder as assessed with the Family Interview for Genetic Studies (Maxwell, 1992)   Inclusion criteria relatives:   1. first-degree relatives with a lifetime occurrence of psychotic symptoms   Exclusion criteria relatives:   1. use of steroid medication 2. current axis I disorder 3. lifetime history of psychotic disorder   Inclusion criteria patients:   1. DSM-IV diagnosis of nonaffective psychotic disorder as assessed with the Comprehensive Assessment of Symptoms and History (Andreasen *et al.*, 1992) or Schedules for Clinical Assessment for Neuropsychiatry version 2.1 (Wing *et al.*, 1990)   Exclusion criteria patients:   1. brain disease 2. history of head injury with loss of consciousness 3. substance-related psychosis 4. psychosis with a known organic cause |
| STRIP  (Lataster *et al.*, 2011a) | N=138  Healthy controls (n=48);  First-degree relative (n=48);  Psychotic disorder (n=42) | |  |
| *(continued)*  **Table S1.** Overview of in- and exclusion criteria of merged studies | | | |
| Study | N |  | |
| ZAPP  (Thewissen *et al.*, 2008) | N=113  Healthy controls (n=38);  Psychotic disorder (n=75) | | Inclusion criteria for all participants:   1. age between 18 and 65 2. sufficient command of the Dutch language   Inclusion criteria controls:   1. average range score on symptom dimensions (between the 45th and 55th percentile) of the Community Assessment of Psychic Experiences (CAPE) (Hanssen *et al.*, 2005)   Exclusion criteria controls:   1. high scores on paranoid items (90^th^ percentile) on the CAPE   Inclusion criteria patients:   1. ICD-10 diagnosis of psychotic disorder as assessed with OPCRIT computer program (McGuffin *et al.*, 1991), PANSS (Kay *et al.*, 1987), and the Life Chart (Susser, 1991) 2. current paranoid and/or positive psychotic symptoms or remitted psychotic symptoms as assessed with the PANSS (Kay *et al.*, 1987) (items P1, P3, P5, P6, and G9) |
| Aripiprazole  (Lataster *et al.*, 2011b) | N=19  Psychotic disorder | | Inclusion criteria patients:   1. age between 18 and 65 2. sufficient command of the Dutch language 3. DSM-IV diagnosis of schizophrenia as generated with the OPCRIT computer program (McGuffin *et al.*, 1991) 4. insufficient therapeutic response to antipsychotic treatment 5. current use of a traditional dopamine antagonist antipsychotic   Exclusion criteria patients:   1. hospitalization within 2 month prior to study 2. endocrine, cardiovascular, or brain disease; history of neuroleptic malignant syndrome 3. pregnancy or lactation (in women) |

| **Table S2.** Model fit statistics for auto-regressive and cross-lagged panel mediation models | | | | | | |
| --- | --- | --- | --- | --- | --- | --- |
|  | | **Model fit statistics** | | | | |
|  | | **LL** | **FP** | **AIC** | **BIC** | **SABIC** |
| Auto-regressive model^a^ | | -46268.60 | 39 | 92615.21 | 92926.22 | 92802.28 |
| Cross-lagged panel model (Momentary stress → negative affect → psychotic experiences) | | -46149.10 | 51 | 92400.20 | 92806.91 | 92644.83 |
| Cross-lagged panel model (Psychotic experiences → negative affect → momentary stress) | | -46151.44 | 51 | 92404.87 | 92811.58 | 92649.50 |
| Combined cross-lagged panel model^b^ | | -45710.46 | 117 | 91654.98 | 92587.95 | 92216.13 |
| *Note.* LL=Log-Likelihood, FP=Free Parameters; AIC=Aikaike Information Criterion; BIC=Bayesian Information Criterion; SABIC= Sample-Size Adjusted Bayesian Information Criterion; ^a^ Model includes pathway from momentary stress to psychotic experiences, and vice versa; ^b^ Model includes longitudinal pathway from momentary stress to psychotic experiences via negative affect, and vice versa. | | | | | | |
|  |  |  |  |  |  |  |

**Andreasen, N. C., Flaum, M. & Arndt, S.** (1992). The Comprehensive Assessment of Symptoms and History (CASH). An instrument for assessing diagnosis and psychopathology. *Arch Gen Psychiatry* **49**, 615-23.

**Collip, D., Nicolson, N. A., Lardinois, M., Lataster, T., van Os, J., Myin-Germeys, I. & G.R.O.U.P** (2011). Daily cortisol, stress reactivity and psychotic experiences in individuals at above average genetic risk for psychosis. *Psychol Med* **41**, 2305-15.

**Hanssen, M., Bak, M., Bijl, R., Vollebergh, W. & van Os, J.** (2005). The incidence and outcome of subclinical psychotic experiences in the general population. *Br J Clin Psychol* **44**, 181-91.

**Kay, S. R., Fiszbein, A. & Opfer, L. A.** (1987). The positive and negative syndrome scale (PANSS) for schizophrenia. *Schizophrenia bulletin* **13**, 261.

**Lataster, J., Collip, D., Ceccarini, J., Haas, D., Booij, L., van Os, J., Pruessner, J., Van Laere, K. & Myin-Germeys, I.** (2011a). Psychosocial stress is associated with in vivo dopamine release in human ventromedial prefrontal cortex: a positron emission tomography study using [(1)(8)F]fallypride. *Neuroimage* **58**, 1081-9.

**Lataster, J., Myin-Germeys, I., Wichers, M., Delespaul, P. A., van Os, J. & Bak, M.** (2011b). Psychotic exacerbation and emotional dampening in the daily life of patients with schizophrenia switched to aripiprazole therapy: a collection of standardized case reports. *Ther Adv Psychopharmacol* **1**, 145-51.

**Maxwell, M. E.** (1992). Family Interview for Genetic Studies (FIGS): a manual for FIGS. *Clinical Neurogenetics Branch, Intramural Research Program, National Institute of Mental Health, Bethesda, MD*.

**McGuffin, P., Farmer, A. & Harvey, I.** (1991). A polydiagnostic application of operational criteria in studies of psychotic illness. Development and reliability of the OPCRIT system. *Arch Gen Psychiatry* **48**, 764-70.

**Myin-Germeys, I., van Os, J., Schwartz, J. E., Stone, A. A. & Delespaul, P. A.** (2001). Emotional reactivity to daily life stress in psychosis. *Arch Gen Psychiatry* **58**, 1137-44.

**Susser, M.** (1991). What is a cause and how do we know one? A grammar for pragmatic epidemiology. *Am J Epidemiol* **133**, 635-48.

**Thewissen, V., Bentall, R. P., Lecomte, T., van Os, J. & Myin-Germeys, I.** (2008). Fluctuations in self-esteem and paranoia in the context of daily life. *J Abnorm Psychol* **117**, 143-53.

**van der Steen, Y., Gimpel-Drees, J., Lataster, T., Viechtbauer, W., Simons, C. J. P., Lardinois, M., Michel, T. M., Janssen, B., Bechdolf, A., Wagner, M. & Myin-Germeys, I.** (2017). Clinical high risk for psychosis: the association between momentary stress, affective and psychotic symptoms. *Acta Psychiatr Scand* **136**, 63-73.

**Wing, J. K., Babor, T., Brugha, T., Burke, J., Cooper, J. E., Giel, R., Jablenski, A., Regier, D. & Sartorius, N.** (1990). SCAN. Schedules for Clinical Assessment in Neuropsychiatry. *Arch Gen Psychiatry* **47**, 589-93.

**Wittchen, H. U., Jacobi, F., Rehm, J., Gustavsson, A., Svensson, M., Jonsson, B., Olesen, J., Allgulander, C., Alonso, J., Faravelli, C., Fratiglioni, L., Jennum, P., Lieb, R., Maercker, A., van Os, J., Preisig, M., Salvador-Carulla, L., Simon, R. & Steinhausen, H. C.** (2011). The size and burden of mental disorders and other disorders of the brain in Europe 2010. *Eur Neuropsychopharmacol* **21**, 655-79.
